# Repression of bacterial gene expression by antivitamin B_12_ binding to a cobalamin riboswitch

**DOI:** 10.1039/d5cb00308c

**Published:** 2026-01-26

**Authors:** Florian J. Widner, Naziyat I. Khan, Evelyne Deery, Martin J. Warren, Michiko E. Taga, Bernhard Kräutler

**Affiliations:** a Institute of Organic Chemistry & Center for Molecular Biosciences (CMBI), University of Innsbruck 6020 Innsbruck Austria bernhard.kraeutler@uibk.ac.at; b School of Biosciences, University of Kent Canterbury CT2 7NJ UK; c Quadram Institute Bioscience, Norwich Research Park Norwich NR4 7UA UK martin.warren@quadram.ac.uk; d Department of Plant and Microbial Biology, University of California Berkeley California USA taga@berkeley.edu

## Abstract

The *E. coli btuB* riboswitch is a cobalamin-sensing RNA element that selectively binds coenzyme B_12_ (adenosylcobalamin, AdoCbl) to downregulate the expression of the outer membrane B_12_-transporter BtuB. Here, we examined adenosylrhodibalamin (AdoRhbl), the isostructural Rh-analogue of AdoCbl, as a surrogate effector ligand for this riboswitch. Two riboswitch-reporter systems were employed: an engineered *E. coli* strain with a fluorescent reporter for intracellular AdoCbl-sensing, and a plasmid-based construct for analogous *in vitro* transcription/translation assays. In the *in-vitro* system AdoRhbl closely mimicked AdoCbl in down-regulating reporter expression with apparent EC_50_ values of 2.8 µM and 0.8 µM respectively. In contrast, the engineered *E. coli* strain revealed much higher effective sensitivities, with EC_50_ values of 1.4 nM for AdoRhbl and of 6.9 nM for AdoCbl, reflecting strong intracellular accumulation of both corrinoids, and comparably efficient uptake. These findings uncover a previously undocumented gene-regulatory activity of an antivitamin, suggesting that AdoRhbl can repress bacterial B_12_ uptake by binding to the *btuB* riboswitch. Together with its ability to inhibit AdoCbl-dependent enzymes, the designed antivitamin B_12_AdoRhbl thus emerges as a multifunctional antibiotic candidate targeting B_12_-utilizing microorganisms.

## Abstract 2

Coenzyme B_12_ and its Rh-analogue, the antivitamin B_12_AdoRhbl, bind both the *btuB*-riboswitch with exceptional affinity and inhibit expression of the bacterial B_12_ uptake protein *BtuB*, signifying AdoRhbl's high potential as multifunctional antibiotic.

## Graphical abstract

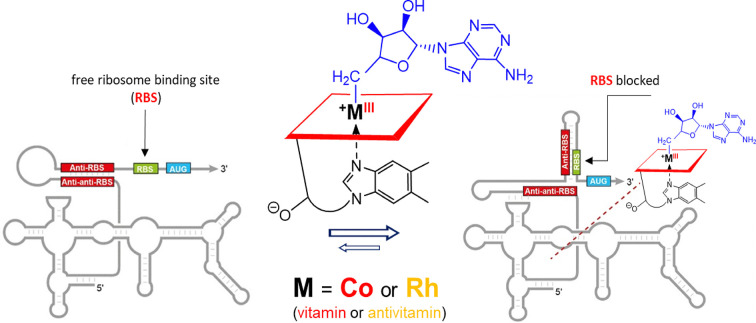

## Introduction

Genetic regulation by bacterial riboswitches, first discovered at the turn of this century,^1,2^ has opened a valuable biological toolbox for controlling transcription, translation, and RNA splicing in microorganisms.^3^ Riboswitches are widespread regulatory mRNA elements that typically consist of two functional domains: an aptamer domain that selectively binds a cognate metabolite, and an expression platform that undergoes a structural rearrangement in response to ligand binding, thereby modulating gene expression.^4^ The first riboswitch identified was the cobalamin-responsive *btuB* riboswitch of *Escherichia coli* (*E. coli*), which binds coenzyme B_12_ (adenosylcobalamin, AdoCbl) with high selectivity,^1^ hence typified as a class-I cobalamin (Cbl) riboswitch.^5^ AdoCbl-binding triggers a conformational rearrangement that sequesters the ribosomal binding site, thereby repressing translation of the outer membrane B_12_-uptake protein BtuB.^6^ In-line probing demonstrated that the 202-nucleotide-long *btuB* riboswitch undergoes this structural shift not only in the presence of AdoCbl but also with vitamin B_12_ (cyanocobalamin, CNCbl), albeit with markedly reduced affinity for CNCbl.^7^ Class-II Cbl-riboswitches, in contrast, preferentially recognise Cbl derivatives with less bulky ‘upper’ axial ligands, such as methylcobalamin (MeCbl).^8^ Moreover, some riboswitches can also sense cobamides (Cbas) other than the canonical Cbls, thus Cbl-riboswitches may be considered an important subgroup of the wider class of B_12_ riboswitches^7^ or corrinoid riboswitches.^9^

Genetic regulation through B_12_ riboswitches in many bacteria reflects their fundamental dependence on the complex natural cobamides as indispensable biocatalyst molecules.^10–14^ Only a subset of these microorganisms possess the complete B_12_-biosynthetic pathway for vitamin B_12_,^15^ while others rely on external sources and scavenge corrinoids from the environment.^16–19^ In order to adjust its metabolism to the availability of extracellular cobalt-corrinoids, *E. coli* employs a B_12_-responsive regulatory system^20^ centred on the *btuB* riboswitch.^1^ This riboswitch plays a vital function by controlling expression of the outer membrane transporter BtuB, which mediates B_12_ uptake.^21,22^

Our interest in the structural basis of *btuB* riboswitch selectivity for corrinoid ligands^7,9^ was been renewed by recent studies on the biological effects of antivitamins B_12_ (*a*VitB_12_s).^23–25^ These compounds were originally designed as metabolically inert structural mimics of vitamin B_12_, *i.e.*, as type-I *a*VitB_12_s,^23^ to induce functional B_12_-deficiency in humans and other mammals, by impairing the cellular supply of active B_12_-cofactors.^26^ In fact, we also anticipated type-I *a*VitB_12_s to impair a broad spectrum of B_12_-dependent cellular processes and to act as bacterial growth inhibitors,^27,28^ like some other B_12_-antimetabolites.^29–31^

**Fig. 1:**
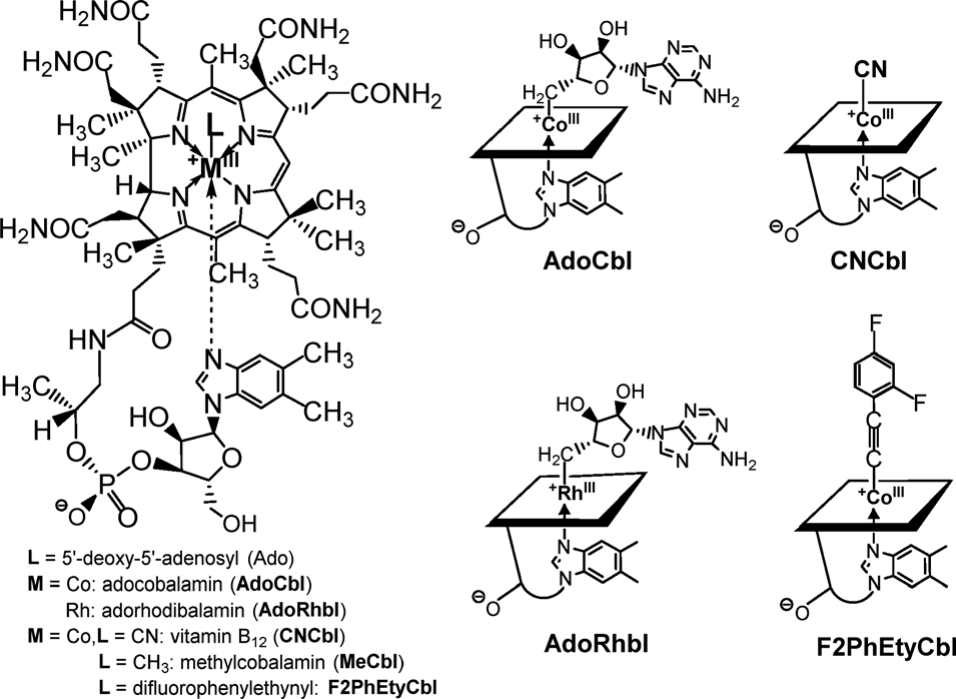
Structural formula (left) and symbols (right, top) of coenzyme B_12_ (adenosylcobalamin, AdoCbl) and vitamin B_12_ (CNCbl), and (right, bottom) of the antivitamins B_12_ adenosylrhodibalamin (AdoRhbl) and 2(2,4-difluorophenyl)ethynylcobalamin (F2PhEtyCbl).

Among the *a*VitB_12_s, the Rh-analogue of AdoCbl, adenosylrhodibalamin (AdoRhbl, Fig. 1), stands out, as it is a nearly perfect structural mimic of AdoCbl^25^ that lacks its essential chemical reactivity.^28,32^AdoRhbl not only resists the critical tailoring catalysed by the (human) enzyme CblC,^32^ but also potently inhibits bacterial AdoCbl-dependent diol-dehydratase,^25^ as well as bacterial and human adenosyltransferases.^32^ It also acts as a light-stable anti-photoregulatory ligand of the widely occurring bacterial CarH photoreceptors.^33^ The particular activity of AdoRhbl as an effective growth-repressor of *Salmonella typhimurum* has raised the question of whether this involves gene regulation *via* a Cbl-riboswitch,^25^ a mechanism proposed as a central mode of activity of proper type-I *a*VitB_12_s.^28^ Because AdoRhbl closely reproduces the molecular shape and surface functionalities of AdoCbl, it is expected to match the natural coenzyme in binding very tightly to class-I Cbl-riboswitches, and in inducing the same conformational changes and downstream regulatory effects. In fact, binding of AdoRhbl to the *btuB*-riboswitch would provide a false-positive signal of intracellular AdoCbl availability, leading to repression of BtuB production, and thereby blocking import of natural B_12_-derivatives, effectively inducing growth-inhibiting Cbl-deficiency.^28^ A natural antibiotic, named roseoflavin, plays a related role by binding flavine-mononucleotide (FMN) riboswitches^34^ and inhibiting bacterial growth.^35^

## Results and discussion

We report here a comparison of the *a*VitB_12_AdoRhbl^25^ and of the natural effector ligand AdoCbl in their capacity of binding to the *btuB* riboswitch and regulating protein expression. For this investigation, we employed two RNA AdoCbl-reporter constructs derived from the *E. coli btuB*-riboswitch.^1,6^ Both constructs (see SI) were designed to provide fluorescence readouts in response to riboswitch-mediated regulation by B_12_-type ligands. Because the isolated (originally used) 240 nt *btuB* riboswitch sequence alone proved insufficient to drive reporter gene expression^20,36,37^ the constructs used here included the promoter, the aptamer, the expression platform and the first 70 codons (210 nucleotides) of the *btuB* coding sequence. Fluorescent protein reporters, red fluorescent protein (RFP) or enhanced green fluorescent protein (eGFP)^38^ were fused downstream. The constructs were incorporated either into the genome of an engineered *E. coli* strain^9^ for *in-vivo* sensing of AdoCbl, or within a correspondingly constructed *E. coli* plasmid, designed for *in-vitro* assays monitoring fluorescence.

**Fig. 2:**
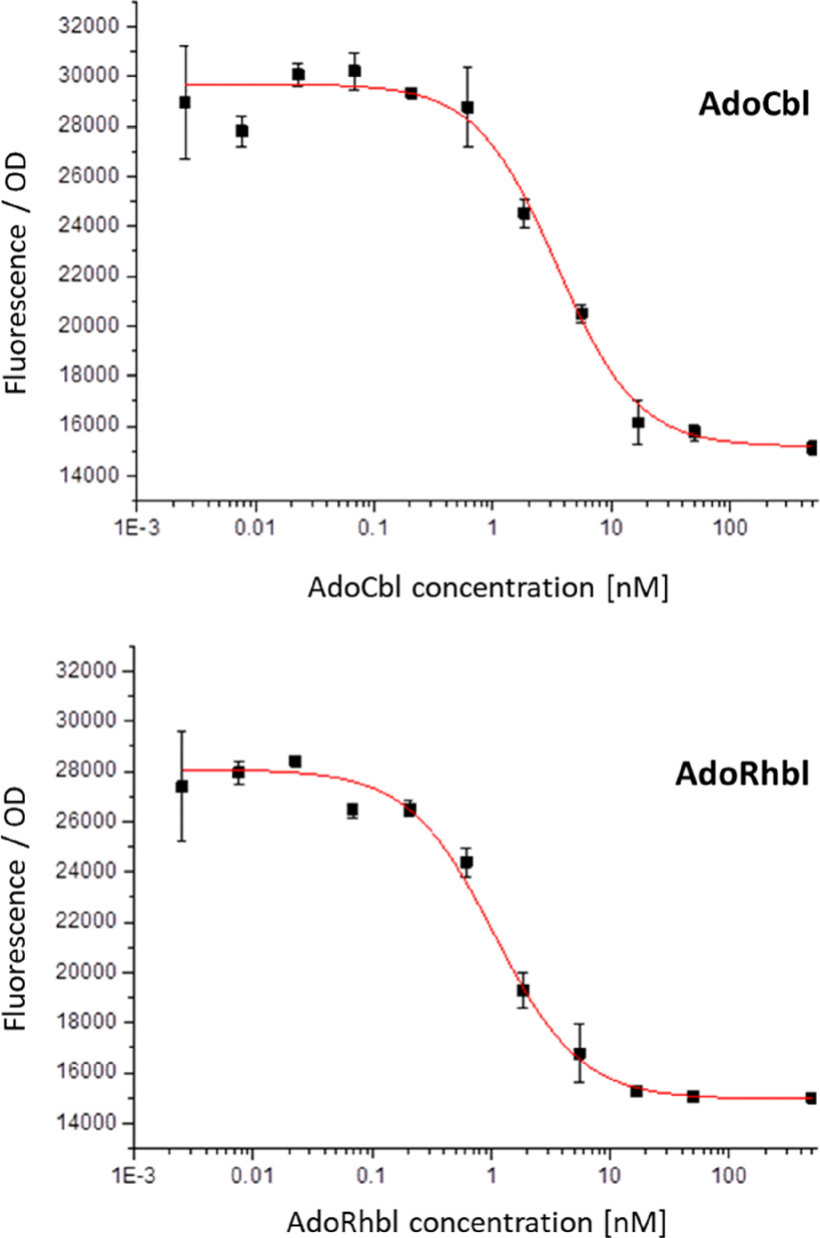
Red fluorescent protein (RFP) fluorescence emission of *E. coli* MG1655 pUC19-BtuB-RFP strain as function of the concentration of added coenzyme B_12_ (AdoCbl, top) or of added adenosylrhodibalamin (AdoRhbl, bottom) with excitation at 530 nm and emission at 590 nm (error bars signify magnitude of st_dev_). Fluorescence readout for AdoCbl indicated an EC_50_ = 6.9 nM (st_dev_ = 4nM); for AdoRhbl an EC_50_ = 1.4 nM (st_dev_ = 0.6 nM) was determined.

The *a*VitB_12_AdoRhbl^25^ was produced for these studies *via* a one-step synthesis from chlororhodibalamin.^39^ As shown here, AdoRhbl acts as a highly potent and specific ligand for the AdoCbl-sensitive *btuB* riboswitch. To investigate intracellular sensing of AdoCbl and AdoRhbl, we employed *E.coli* MG1655 harboring a pUC19 plasmid containing a BtuB-RFP fusion (pUC19-BtuB-RFP),^9^ in which RFP expression is regulated by the *btuB* B_12_-riboswitch (for details, see SI).^40^ Bacterial cultures were diluted and exposed to either AdoCbl or AdoRhbl, over a concentration range from 2.54 pM to 0.5 µM. Upon light-protected incubation for 16 h at 37 °C, RFP-fluorescence was determined at 590 nm. In both cases, fluorescence decreased in a concentration-dependent manner, with reproducible results in two independent experimental series. These data indicate efficient binding of the riboswitch by either corrin, with a critical transition observed at low nM concentrations (Fig. 2).

Curve fitting with a 1 : 1 binding isotherm model provided effective EC_50_s of 1.4 nM (+/−0.6 nM) for the Rh-corrin AdoRhbl and 6.9 nM (+/−4 nM) for the homologous Co-corrin AdoCbl. Both the natural B_12_-cofactor AdoCbl and its isostructural Rh-analogue AdoRhbl efficiently triggered switching of the *btuB*-riboswitch construct at remarkably low nM concentrations in the medium. The high intracellular sensitivity of this class-I Cbl-riboswitch to either organometallic Ado-corrinoid is consistent with the well-documented extensive accumulation of AdoCbl in bacteria,^40,41^ and is matched by the designed Rh-corrin AdoRhbl. Notably, the conformational switch induced by AdoRhbl occurs at roughly 5-fold lower extracellular concentrations than with the natural cognate ligand AdoCbl. This apparent difference does not reflect a higher intrinsic binding affinity of the riboswitch for the *a*VitB_12_AdoRhbl, which was not observed in *in-vitro* experiments (see below). Rather, it likely arises because a fraction of the internalized AdoCbl is diverted into metabolism, serving as a precursor for other Cbls, such as methylcobalamin (MeCbl),^41^ which bind the class-I Cbl- riboswitch *btuB* with lower affinity.^42^

The strong intracellular sensing of AdoCbl and AdoRhbl by the *btuB* riboswitch encouraged us to examine binding properties of a more readily accessible and robust Cbl-based *a*VitB_12_, the alkynylcobalamin 2(2,4-difluorophenyl)ethynyl-cobalamin (F2PhEtyCbl)^43^ (see SI). At solution concentrations above 10 nM, F2PhEtyCbl reproducibly reduced fluorescence in *E. coli* cells carrying the pUC19-BtuB-RFP construct, consistent with riboswitch downregulation of RFP expression. Data fitting over the concentration range of 0.2 nM to >10^4^ nM yielded an apparent EC_50_ of about 180 nM (see SI, Fig. S1). The roughly 100-fold weaker riboswitch binding affinity of F2PhEtyCbl relative to AdoCbl or AdoRhbl reflects the known sensitivity of class-I Cbl- riboswitches to the structure of the cofactor's ‘upper’ ligand,^7^ and is in line with the reported lack of evidence for effective binding of structurally related alkynylcobalamins to the *E. coli btuB*-riboswitch.^44^

In the alternative ‘*in-vitro’* experiments with the *E.coli btuB* riboswitch a corresponding test system based on an *E. coli in-vitro* transcription/translation kit was used (see SI). A key advantage of this ‘*in-vitro’* setup is that it directly reports on interactions between the riboswitch and the effector corrinoids, thereby allowing determination of the riboswitch response to the solution concentration of the supplied ligand. In the context of this study, these data provided a basis for estimating how intracellular accumulation of corrinoids influences *in vivo* riboswitch response observed in the engineered *E. coli* strain.

For the ‘*in-vitro’* system, the riboswitch construct was generated within a pET14b plasmid carrying the gene for eGFP under the control of a T7 promoter. Using specific primers, the functional *btuB* riboswitch segment was PCR amplified and inserted upstream of the eGFP coding sequence, yielding Rib70-*eGFP*-pET14b (described in the SI, Fig. S2 and Table S1). This design positioned the riboswitch correctly in-frame between the T7 promoter and the *eGFP* reporter gene, enabling riboswitch-dependent regulation of eGFP expression.

**Fig. 3:**
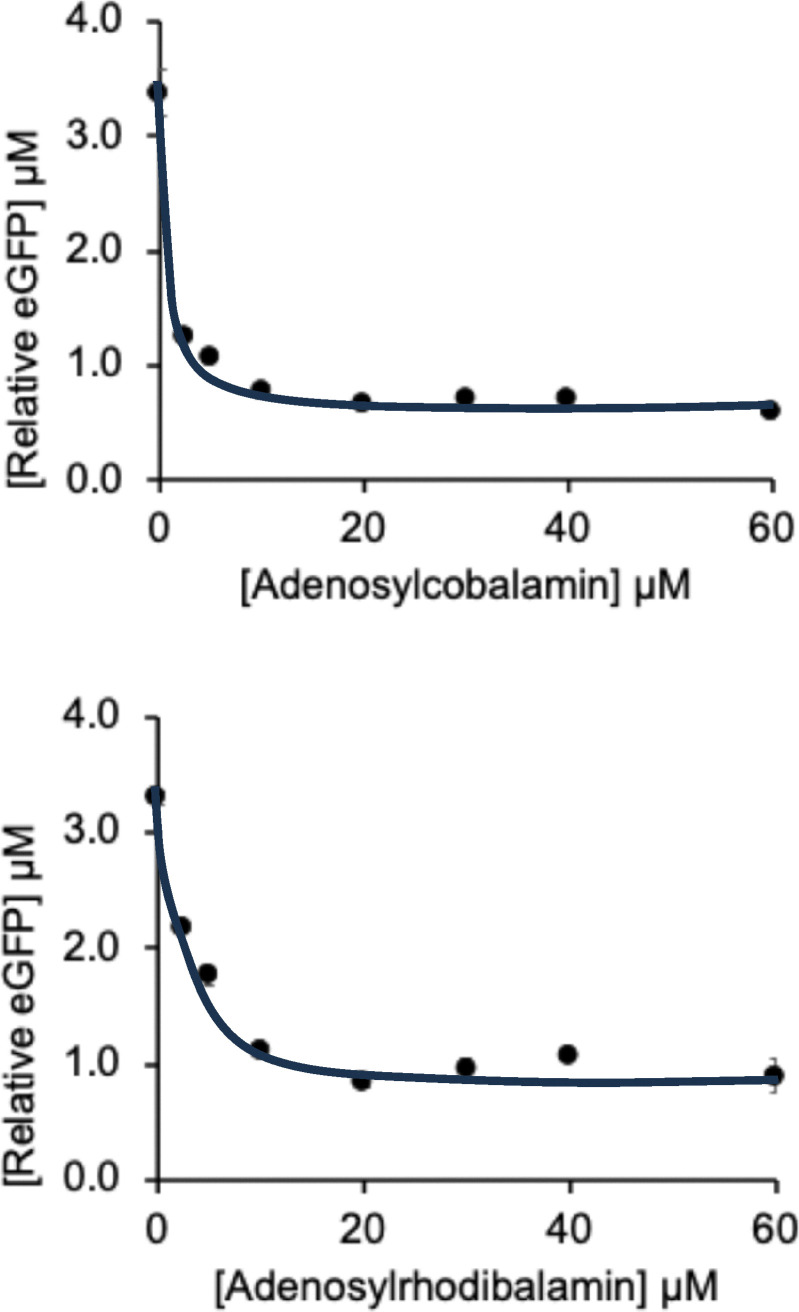
Graphs showing the effect of increasing AdoCbl (top) and AdoRhbl (bottom) concentration on *btuB* riboswitch-controlled eGFP-production as analyzed by its fluorescence (excitation at 488 nm; detection at 510 nm). From curve-fitting, using 1 : 1 binding isotherm models, for AdoCbl an EC_50_ value of 0.8 µM (+/−0.1 µM) was estimated, for AdoRhbl an EC_50_ value of 2.8 µM (+/−0.7 µM).

To confirm that Rib70-*eGFP*-pET14b supported reporter expression, the plasmid was transformed into *E. coli* BL21*(DE3)-pLysS and cultured in M9 minimal medium. The resulting cell pellets appeared green, demonstrating successful eGFP production. To assess riboswitch control and activity in response to AdoCbl, *in vitro* protein synthesis experiments were performed using the NEB PURExpress transcription/translation kit (see SI). Riboswitch function was evaluated by quantifying eGFP production, both *via* fluorescence intensity measurements (Fig. 3), and by densitometry of western blots (SI, Fig. S3). To examine the regulatory response to AdoCbl and AdoRhbl, *in vitro* reactions were conducted across a ligand concentration range of 0-60 µM. The resulting values were analyzed by curve-fitting using 1 : 1 isotherm models (Fig. 3 and SI Fig. S3).

Since both AdoCbl and AdoRhbl are colored at higher concentrations, their potential interference with eGFP fluorescence was examined. Calibration curves were generated by recording eGFP fluorescence at known concentrations in the presence of varying amounts of AdoCbl or AdoRhbl. Comparable fluorescence intensities were obtained in samples with and without corrinoids, confirming that neither ligand significantly affected the eGFP fluorescence signal.

Calibration curves generated with defined concentrations of AdoCbl were used to relate fluorescence intensities to relative eGFP levels in each *in vitro* reaction. To validate these measurements, western blotting followed by densitometric analysis was performed, comparing band intensities of the reaction samples with eGFP standards of known concentration. This dual approach enabled accurate determination of relative eGFP levels across the samples. The combined fluorescence and densitometry datasets were then used to plot the effect of increasing AdoCbl or AdoRhbl concentrations on eGFP production under *btuB* riboswitch control (Fig. 3 and SI, Fig. S3).

Our findings demonstrate that both AdoCbl and AdoRhbl bind to and repress the *btuB* riboswitch, as evidenced by a concentration dependent decrease in eGFP production between 2.5 µM to 60 µM. The similar response profiles indicate that AdoCbl and AdoRhbl exhibit comparably strong binding affinities for the riboswitch, which is consistent with their close structural similarity.^25^ Interestingly, neither ligand completely switches off the riboswitch activity: even at 60 µM ligand concentration, residual eGFP production persisted. Quantitative analysis suggests that both AdoCbl and AdoRhbl reduce translation by about 80% (SI Table S2). This incomplete repression likely reflects suboptimal folding of this structurally complex riboswitch in the course of AdoCbl binding, permitting constitutive low-level protein production, presumably a striking physiological safeguard against Cbl-deficiency.

To confirm that the observed reduction in eGFP production was specifically mediated through riboswitch binding, rather than interference with the *in vitro* translation system, we employed a control plasmid (*eGFP*-pET14b) lacking the riboswitch sequence. Under identical conditions, eGFP expression from this construct was unaffected by either AdoCbl or AdoRhbl, confirming that both ligands act solely through riboswitch-dependent regulation.

**Fig. 4:**
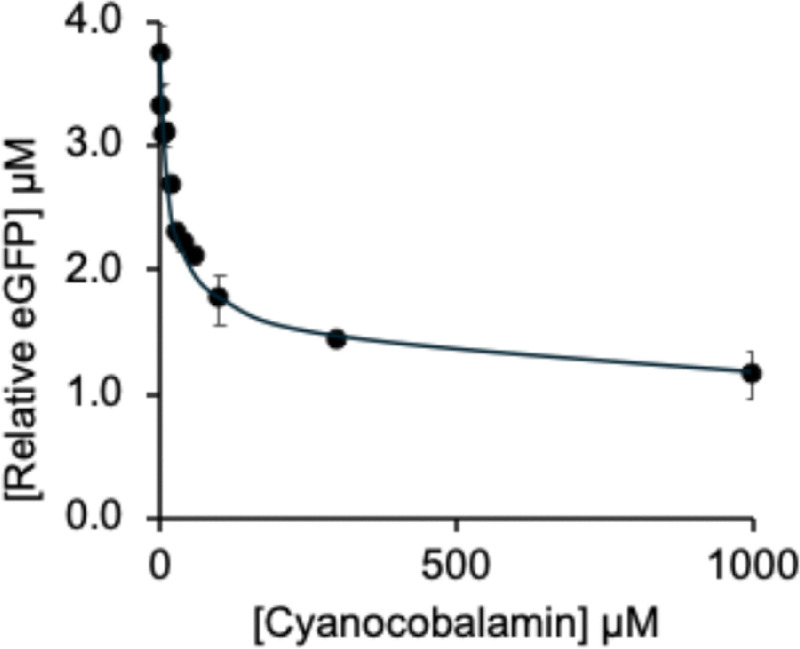
Graph showing the effect of increasing CNCbl concentration on *btuB* riboswitch-controlled eGFP production, monitored by fluorescence analysis at 510 nm. From curve-fitting, using a 1 : 1 binding isotherm model, an EC_50_ value of 31.4 µM (+/−5.2 µM) was estimated.

The plasmid Rib70-eGFP-pET14b carrying the “full-length” *btuB* riboswitch was also employed for *in vitro* assays with vitamin B_12_ (CNCbl). These experiments confirmed CNCbl binding to the *btuB* riboswitch, leading to reduced eGFP expression, as reflected in the concentration-dependent decline in fluorescence (Fig. 4 and SI, Fig. S4). However, CNCbl repressed expression only partially, with fluorescence measurements indicating a maximum deactivation of about 63% (SI, Table S2). Half-maximal deactivation was reached at 31.4 µM according to fluorescence data, and at 77.3 µM based on densitometric analysis. These results demonstrate that CNCbl has a lower affinity for the *btuB* riboswitch and does not achieve the same level of repression as AdoCbl, in qualitative agreement with earlier in-line probing studies.^7^

Our results show that the *E. coli btuB* B_12_-riboswitch discriminates only minimally between its natural effector ligand AdoCbl and the iso-structural *a*VitB_12_AdoRhbl, in which the Co(iii)-centre of AdoCbl is replaced by Rh(iii).^25^*In vitro* fluorescence and densitometry analyses with the eGFP/*btuB* reporter construct yielded EC_50_ values of 800 nM and 3.1 µM for AdoCbl, and 2.8 µM and 1.9 µM for AdoRhbl, respectively (Fig. 3 and SI, Fig. S3). The nearly identical regulatory effects of AdoCbl and AdoRhbl reflect their close structural similarity, with AdoCbl being the cognate ligand of the class-I Cbl-riboswitch. These EC_50_ values are broadly consistent with *K*_D_-values obtained by in-line probing experiments of the isolated 202 nucleotide *btuB* riboswitch, for which the somewhat lower *K*_D_ values of 300 nM^6^ and 89 nM^7^ were reported, possibly reflecting (in part) differences between mere riboswitch binding and its actual effect on gene-expression.

Strikingly, *in vivo* experiments using an engineered *E. coli* MG1655 strain carrying the intracellular sensor revealed EC_50_ values nearly three orders of magnitude lower: 6.9 nM for AdoCbl and 1.4 nM for AdoRhbl (Fig. 2). These values are comparable to previous measurements with related *E. coli* riboswitch sensors for AdoCbl,^40^ and confirm the highly effective binding of AdoRhbl to the *btuB* riboswitch *in vivo*. The strong intracellular signal induced by externally supplied AdoRhbl indicates that this non-natural B_12_-mimic is imported into bacterial cells with similar efficiency to AdoCbl. The apparent increase in sensitivity *in vivo* likely reflects the substantial intracellular accumulation of Cbls in growing *E. coli*, estimated to reach approximately 1000-fold higher than in the growth medium.^40,41^ Indeed, the similar BtuB-dependent intracellular accumulation of AdoRhbl and AdoCbl, recently documented in both *E.coli* and *Myxococcus xanthus*,^33^ is consistent with the ratios of their deduced *K*_D_-values *in vivo versus in vitro*.

In contrast to AdoRhbl, which binds the *E. coli btuB* riboswitch with affinities comparable to the natural ligand AdoCbl, the more readily available Cbl-based *a*VitB_12_F2PhEtyCbl^43^ exhibits roughly 100 times lower affinity with an intracellular EC_50_ of 180 nM, qualitatively consistent with its ‘*in-vitro*’ EC_50_ of about 340 µM (see SI, Fig. S4b). A similar reduction in affinity is expected for other stable Cbl-based *a*VitB_12_s carrying alternative ‘upper’ axial ligands. Indeed, a hardly detectable *btuB* riboswitch binding has been reported for several such non-natural Cbls,^44^ consistent with the established preference of class-I Cbl-riboswitches for AdoCbl over other natural corrinoids.^1,7^

The precise structural basis for AdoCbl recognition by the *btuB* riboswitch remains unresolved, and high-resolution three-dimensional structures of B_12_-riboswitches are still scarce.^5,8,45,46^ Nonetheless, the crystal structure of a 172 nucleotide class-I Cbl-riboswitch from *Symbiobacterium thermophilum* revealed a B_12_-binding pocket with a well-defined complementarity to the ‘upper’ Ado-ligand, providing a rationale for its strong selectivity towards AdoCbl.^45^ A comparable architecture has been described for the AdoCbl-selective riboswitch from *Thermoanaerobacter tengcongensis*.^8^ By contrast, the env8 riboswitch displays a roughly 10^4^-fold preference for MeCbl over AdoCbl.^42^ Structural analysis showed that its narrower B_12_-binding cavity restricts accommodation of the bulky Ado-group while permitting favourable interactions with smaller ‘upper’ axial ligands.^8,44^ In addition, an ‘atypical’ B_12_-riboswitch from *Bacillus subtilis* demonstrates structural flexibility that allows broader corrinoid recognition, consistent with its more promiscuous ligand-bonding profile.^9,46^

The discovery of natural B_12_-riboswitches that preferentially bind corrinoids with smaller ‘upper’ axial ligands, such as MeCbl and H2OCbl, now typified as class-II Cbl- riboswitches,^8,42,44,46^ suggests that analogous ligand selectivity could be exploited in the design of corresponding *a*VitB_12_s. Guided by structure-based design, Rh-analogues of key Cbls represent promising *a*VitB_12_ candidates.^28^ Indeed, several structurally characterized rhodibalamins (Rhbls), including methylrhodibalamin (MeRhbl),^47^ chlororhodibalamin (ClRhbl)^39^ and acetylrhodibalamin (AcRhbl),^48^ (see SI), represent potential further tools to probe class-II and other Cbl-riboswitches. In parallel, the broader selectivity of ‘promiscuous’ natural B_12_-riboswitches for cobamides (Cbas) with different ‘lower’ ligands^9^ provides an additional design principle for novel *a*VitB_12_s, this time mimicking other Cba structures. Such natural preferences could be harnessed by tailoring Rhbls to exploit the structure-selective binding patterns of these riboswitches. Synthetic access to Rh-analogues of ‘incomplete’ natural corrinoids has provided advanced Rhbl-precursors, such as adenosylrhodibyrate (AdoRhby)^25,32^ and methylrhodibyrate (MeRhby) (see SI).^49^ Remarkably, key B_12_-biosynthetic enzymes exhibit sufficient promiscuity to accept Rh-substituted corrinoid substrates in place of their natural cobalt counterparts.^25,32^ As a result, certain rhodibinamides (Rhbis), such as AdoRhbi,^49^ may serve as substrates for alternative metabolic or enzymatic avenues to generate ‘complete’ Rhbls and other Rhbas featuring an adenosyl ‘upper’ ligand.

As exemplified by AdoRhbl, carefully designed *a*VitB_12_s hold great promise as bacterial growth inhibitors owing to their high affinity for Cbl-riboswitches. Metabolite-sensing riboswitches have recently emerged as novel antibacterial drug targets,^50–52^ where appropriately tailored ligands may offer valuable new approaches to antibiotic development.^53–55^ Given the central roles of B_12_-derivatives in microbial physiology,^10–14^ B_12_-dependent riboswitches represent particularly promising additions to the repertoire of riboswitch-based drug targets.^50,52,56^ In this context, AdoRhbl, a close structural AdoCbl-mimic, illustrates the fundamental potential of rationally designed *a*VitB_12_s as drug candidates,^28^ since AdoCbl-responsive class-I Cbl-riboswitches, such as the *btuB* riboswitch, are key regulators of corrinoid uptake and metabolism in various bacteria.

## Conclusions

Our here described experimental study firmly supports the proposal that a type-I antivitamin can closely mimic the regulatory role as riboswitch ligand of the naturally selected vitamin.^28^ In fact, the *a*VitB_12_AdoRhbl bound the *btuB* riboswitch very tightly and functioned as a highly effective surrogate for the natural effector ligand AdoCbl of this key Cbl-riboswitch. By repressing expression of the B_12_-uptake protein BtuB, monitored here *via* the fluorescence of RFP and eGFP, AdoRhbl can potentially block the external B_12_-supply required for *E. coli* growth. Beyond this riboswitch-mediated control, AdoRhbl acts as a potent *a*VitB_12_^28,32^ and as effective inhibitor of AdoCbl-dependent enzymes,^25,32^ establishing and strengthening its potential as a multifunctional antibiotic that operates through several mutually independent growth-inhibiting mechanisms.^23,28^ Rhodium-based *a*VitB_12_s, designed as precise structural mimics of Cbl-cofactors,^28,47^ may be broadly useful multifunctional suppressors of the essential activities of natural B_12_-derivatives and thereby serve as selective and powerful novel inhibitors of the growth of B_12_-dependent bacteria.

## Author contributions

F. J. W., M. J. W., M. E. T. and B. K. conceived the project. F. J. W., N. I. K., E. D. and M. J. T. designed and performed experiments. All authors discussed the results. F. J. W., M. J. W., M. E. T. and B. K. wrote the manuscript.

## Conflicts of interest

There are no conflicts to declare.

## Supplementary Material

CB-007-D5CB00308C-s001

## Data Availability

The data supporting this article have been included in the supplementary information (SI). Supplementary information is available. See DOI: https://doi.org/10.1039/d5cb00308c.
