# Supplementary material for: Repression of bacterial gene expression by antivitamin B12 binding to a cobalamin riboswitch
Source: RSC Chem Biol. 2026 Jan 26;7(3):498–504. doi: 10.1039/d5cb00308c (PMC12870672; doi:10.1039/d5cb00308c)
Supplement: CB-007-D5CB00308C-s001 [file CB-007-D5CB00308C-s001.pdf]

Supplementary Information (SI) for RSC Chemical Biology.
This journal is © The Royal Society of Chemistry 2026

Repression of bacterial gene expression by antivitamin B_{12} binding to a cobalamin riboswitch

Florian J. Widner[a], Naziyat I. Khan[b], Evelyne Deery[b],

Martin J. Warren[b,c]*, Michiko E. Taga[d]* and Bernhard Kräutler[a]*

[a] Institute of Organic Chemistry & Center for Molecular Biosciences (CMBI), University of Innsbruck, 6020 Innsbruck (Austria)

[b] School of Biosciences, University of Kent, Canterbury, CT2 7NJ (UK)

[c] Quadram Institute Bioscience, Norwich Research Park, Norwich NR4 7UA (UK)

[d] Department of Plant and Microbial Biology, University of California, Berkeley, California (USA)

Supplementary Information

Materials:

Water (H_2O; purified over reverse osmosis); ddH_2O (Millipore MiliQ Academic, HPLC gradient grade); acetonitrile (MeCN; methanol (MeOH) BDH prolabo, >99.9%, HPLC gradient grade). Crystalline adenosylrhodibalamin (**AdoRhbl**) was prepared from chlororhodibalamin (**ClRhbl**),[1] and spectroscopically identified, as described by Widner et al.[1-2] coenzyme B_{12} (**AdoCbl**) from Fluka (>96% purity) was recrystallized from H_2O/acetone; vitamin B_{12} (**CNCbl**, ≥ 98%, from Merck) crystalline difluorophenylethinylcobalamin (**F2PhEtyCbl**) was prepared as in [3]; M9 glucose minimal medium (pH 7.4) contained: 2.6 g/L $(NH_4)_2SO_4$, 1.0 g/L NH_4Cl, 0.5 g/L NaCl, 15.0 g/L $Na_2HPO_4 \cdot 12\ H_2O$, 3.0 g/L KH_2PO_4, 50.0 mg/L $FeCl_3 \cdot 6\ H_2O$, 65.0 mg/L EDTA Na_2, 1.8 mg/L $ZnSO_4 \cdot 7$ H2O, 1.8 mg/L $CuSO_4 \cdot 5$ H2O, 1.2 mg/L $MnSO_4 \cdot$H2O, 1.8 mg/L $CoCl_2 \cdot 6\ H_2O$, 2.0 mM $MgSO_4$, 0.2 mM $CaCl_2$, and 0.3 μM thiamine·HCl, 20 mM glucose as carbon source; components were prepared and sterilized separately and mixed under sterility: (1) phosphates, sodium chloride and ammonium. (2) iron chloride and edta, (3) copper, zinc, cobalt and manganese salts, (4) magnesium salt, (5) calcium salt and (6) glucose (see various discussions in https://www.researchgate.net/post/Why_is_recombinant_Ecoli_not_growing_in_M9_minimal_media); all chemicals were bought from Merck or Fisher Scientific; cloning: Gibson Assembly® Master Mix from NEB, Zyppy™ plasmid Miniprep Kit, Zyppy DNA Clean & Concentrator™-25, DpnI digest kit (NEB), other restriction enzymes and T4 DNA ligase from Promega, PCR using FastStart High Fidelity from Merck, sequencing performed by Genewiz, Azenta Life Sciences; plates for plate reader assay: Costar 3603 (clear bottom, 96 well, sterile).

Intracellular sensing experiments with fluorescence detection of red fluorescent protein (RFP)

Cloning and transformation of the reporter strain (pUC19-BtuB/RFP in MG1655):

The BtuB riboswitch DNA [4] (promoter, riboswitch aptamer, expression platform and 210 nucleotides of the *btuB* coding sequence [5]) was amplified from chromosomal DNA of *E.coli* MG1655 by PCR. The BtuB riboswitch DNA and the RFP DNA [6] were combined and inserted into pUC19 by Gibson assembly, in order to generate a DNA strand with a comparable sequence topology as published [6]. The assembled sequence was confirmed by Sanger sequencing of the inserted DNA. The construct pUC19-BtuB/RFP was transformed into calcium competent MG1655 cells and selected on LB agar containing ampicillin (100 mg/L).

Preparing a culture for the riboswitch fluorescence assay:

A frozen glycerol stock was used to streak *E.coli* MG1655 strain containing pUC19-BtuB/RFP on a plate (LB Agar ampicillin plate, 37 °C). Starting from one colony an overnight culture in M9 glucose media at 37°C was grown until the optical density at 600 nm ($O.D._{600}$) reached 1.1. The culture was diluted 1/100 in M9 glucose. The diluted culture was grown at 37°C until $O.D._{600}$ reached 0.04 (approx. 2 hours).

E. coli fluorescence measurements:

Fluorescence of RFP was measured using a BioTek Synergy 2 plate reader with excitation at 530 nm and emission measured at 590 nm. The samples contained: 96 µl of fresh M9 Media, 100 µl of culture (OD = 0.04), 4 µl of corrinoid solution. The plate was incubated at 37°C for 16 h with lid until the fluorescence was measured. Two experiments were done in triplicate on different days starting from different colonies.

Concentrations in solution medium of tested B_{12}-analogs:

- **AdoCbl** [nM]: 0, $2.54 *10^{-3}$, $7.62*10^{-3}$, $22.9*10^{-3}$, $68.6*10^{-3}$, 0.206, 0.617, 1.85, 5.56, 16.7, 50.0, 500 (see main part Fig. 2, top)
- **AdoRhbl** [nM]: 0, $2.54*10^{-3}$, $7.62*10^{-3}$, $22.9*10^{-3}$, $68.6*10^{-3}$, 0.206, 0.617, 1.85, 5.56, 16.7, 50.0, 500 (see main part Fig. 2, bottom)
- **F2PhEtyCbl** [nM]: 0, 0.305, 0.914, 2.743, 8.23, 24.7, 74.1, 222, 667, $2.00*10^{3}$, $6.00*10^{3}$, $20.0*10^{3}$ (see below, Fig. S1)

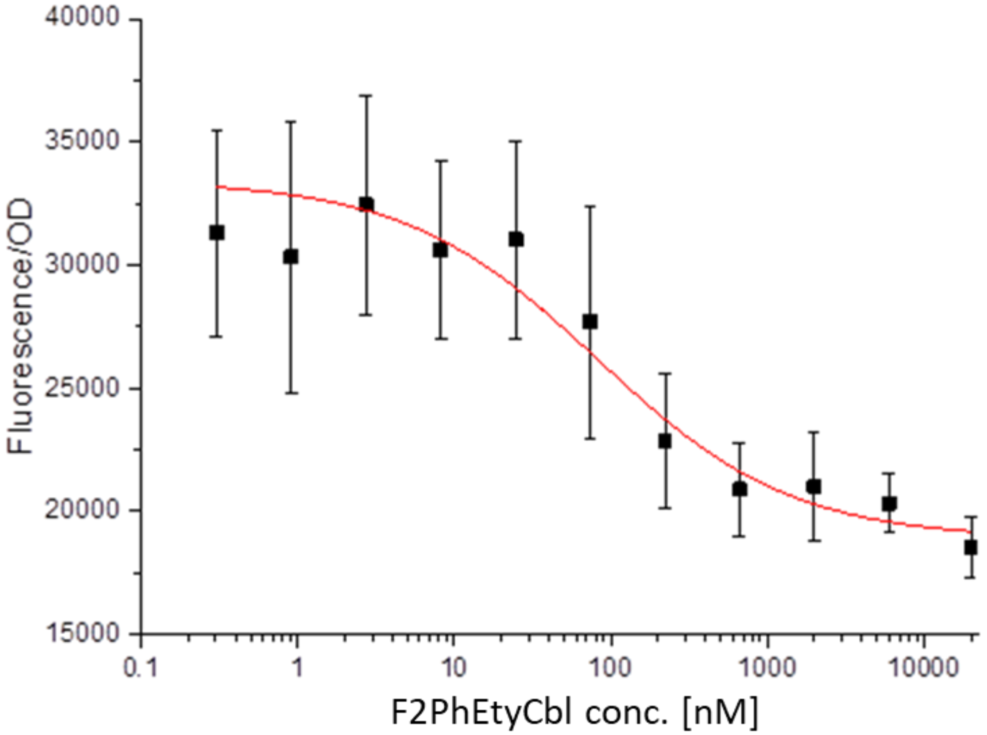

Figure S1. Fluorescence measurement using reporter strain MG1655 containing pUC19-BtuB-RFP and with **F2PhEtyCbl** as ligand, The dependence of the RFP readout on the concentration of the added corrinoid resulted in an EC_{50} = 181 nM (St_{dev} = 139 nM)

'*In-vitro*' experiment with detection of eGFP - procedures and data

Preparation of a riboswitch-controlled reporter gene construct, 'Rib70_eGFP_pET14b'

The *btuB* riboswitch containing the first 70 codons of the *btuB* gene was amplified from *E. coli* K12 MG1655 genomic DNA using the Rib_For and Rib70_Rev primers, synthesized by Invitrogen (Table S1). The PCR-amplified sample was run on a 1% agarose gel, the appropriate approximate 460 bp band was extracted and the DNA was purified from the agarose, before digesting with XbaI/NdeI restriction enzymes. The use of these particular restriction enzymes allows the fragment to be cloned downstream of the T7 promoter in the pET14b vector that housed an eGFP (eGFP-pET14b), before the start codon of the eGFP reporter gene. A ligation between the XbaI/NdeI digested riboswitch (insert) and XbaI/NdeI digested eGFP-pET14b (vector) DNA was furnished the complete plasmid, called Rib70_eGFP_pET14b (Figure S2).

Cloning strategy for the construction of the Rib70_eGFP_pET14b plasmid

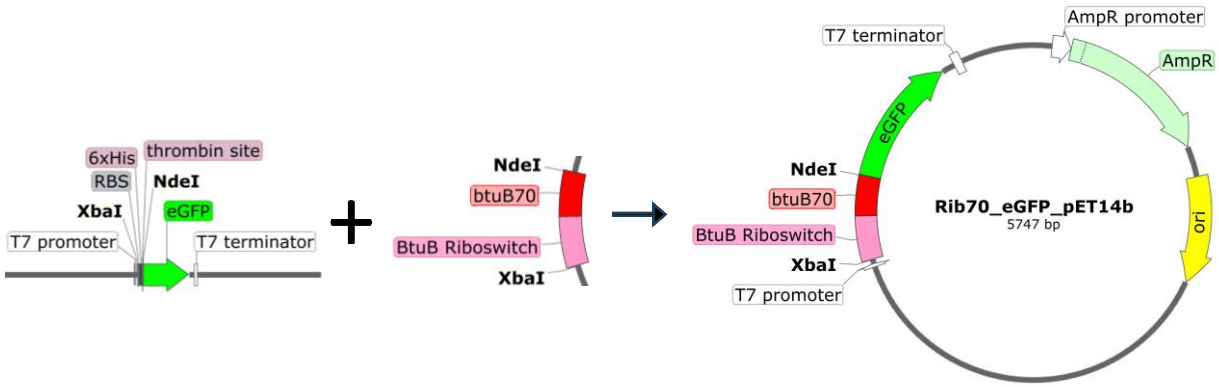

Figure S2. Cloning strategy for the construction of the Rib70_eGFP_pET14b plasmid (SnapGene was used to make this figure). Insertion of the amplified riboswitch fragment (Riboswitch + btuB70) into *eGFP*-pET14b was carried out using XbaI and NdeI restriction sites. Rib70_*eGFP*_pET14b contains Riboswitch-btuB70 upstream of the *eGFP* reporter gene under the control of a T7 promoter and terminator. The plasmid also contains an ampicillin resistance gene, *AmpR* and its promoter, AmpR promoter, for selection purposes.

Table S1. Sequences used here for the Rib_For and Rib70_Rev primers

Rib_For	GCT TCT AGA GCC GGT CCT GTG AGT TAA TAG G	XbaI
Rib70_Rev	GAT CAT ATG CGG AAG ACG GCG CAG CAC	NdeI

In vitro transcription/translation using the PURExpress (#E6800, New England Biolabs) *in vitro* protein synthesis kit

Plasmid DNA samples were purified following the Qiagen minipreps kit protocol, except that they were eluted in 25 µL of 10 mM Tris-Cl at pH 8.5 instead of 50 µL. The lower elution volume allowed the DNA samples to be more concentrated. The concentration was measured on a NanoVue spectrophotometer (GE Healthcare).

Reactions were performed as described in the NEB manual (https://www.neb.com/en-gb/-/media/nebus/files/manuals/manuale6800_e3313_e6840_e6850.pdf?rev=60ba1e078b384d21bf0d5bbfd00495b9&hash=961278C162D92975A553D135BEDD3CCC).

Briefly, the following reaction was assembled on ice: 10 µl of solution A, 7.5 µl of solution B, either 2.5 µl of nuclease-free water or 2.5 µl of B_{12}/B_{12} variant and 5µl of plasmid DNA at 150 ng/µl. The reaction was incubated at 37˚C for 4 hours and finally stopped by transferring the tube on ice. 5 µL of reaction mix was then loaded onto a 12.5% SDS PAGE or was analysed by fluorescence.

Quantification of eGFP by densitometry

The SDS-PAGE was subjected to western blot analysis using the Mouse anti-GFP antibody (Merck) as primary antibody and the Anti-mouse IgG (H+L), AP Conjugate (Promega) as secondary antibody. The Alkaline phosphatase activity was revealed using 5-bromo-4-chloro- 3-indolyl phosphate/nitro blue tetrazolium (BCIP/NBT) (Merck). The eGFP concentrations were analyzed by densitometry of eGFP production (see Fig. S3). Four concentrations of eGFP (4 µM, 2 µM, 1 µM and 0.5 µM) were also subjected to western blot analysis to generate a calibration curve. Densitometry analysis was carried out using ImageJ software.

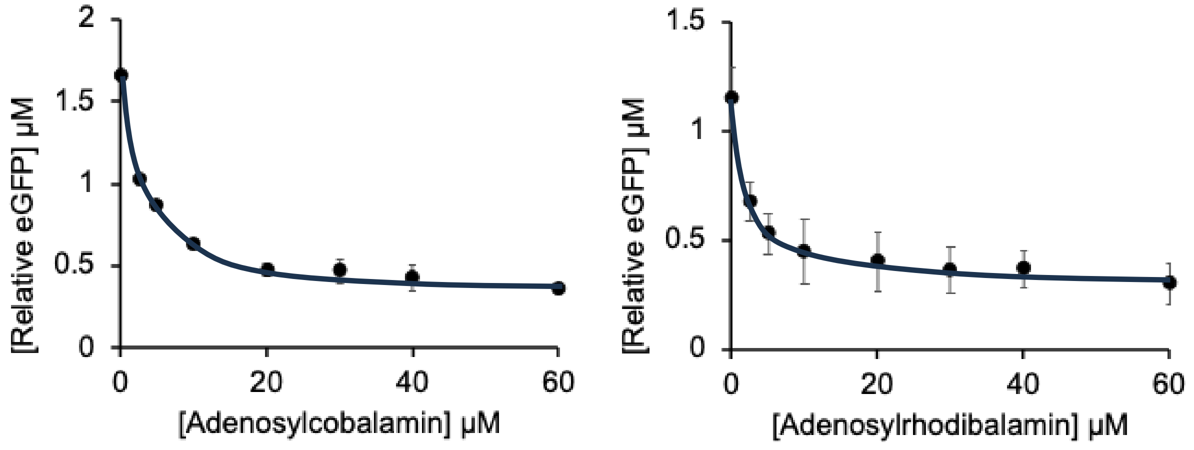

Fig. S3. Graphs showing the effect of increasing **AdoCbl** concentration (left) and **AdoRhbl** concentration (right) on *btuB* riboswitch-controlled eGFP-production, analyzed by densitometry. From curve-fitting, using 1:1 binding isotherm models, For **AdoCbl**, an EC_{50} value of 3.1 µM (+/- 0.3 µM) was estimated, for **AdoRhbl** an EC_{50} value of 1.9 µM (+/- 0.2 µM).

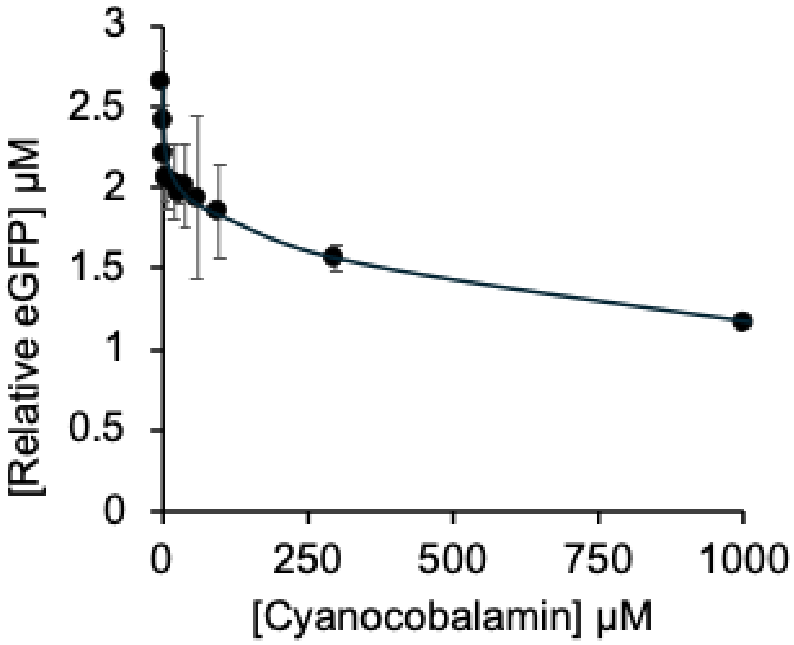

Fig. S4a. Graph showing the effect of increasing **CNCbl** concentration on *btuB* riboswitch-controlled eGFP production, monitored by densitometry. From curve fitting, using 1:1 binding isotherm models, an EC_{50} value of 77.3 µM (+/- 39.2 µM) was determined for **CNCbl**.

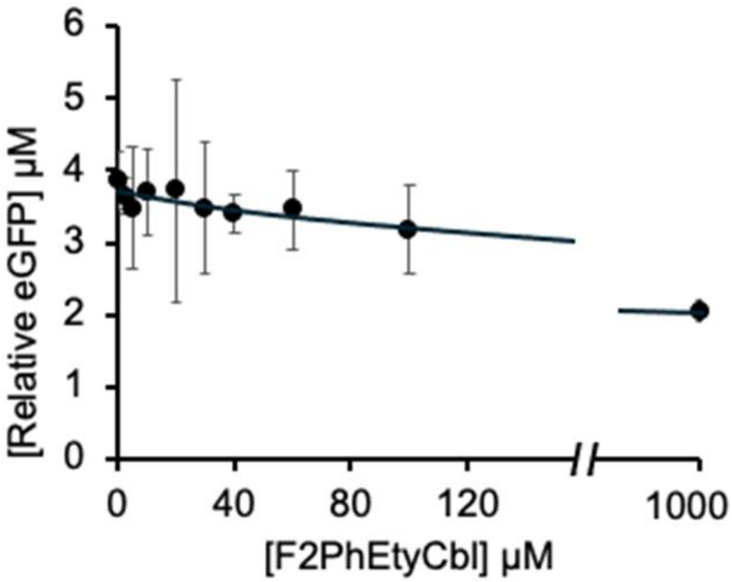

Fig. S4b. Graph showing the effect of increasing **F2PhEtyCbl** concentration on *btuB* riboswitch-controlled eGFP production, monitored by densitometry. From curve fitting, using 1:1 binding isotherm models, an EC_{50} value of 340 µM was estimated for **F2PhEtyCbl**.

Quantification of eGFP by fluorescence

eGFP was directly measured by the fluorescence emitted at 510 nm after an excitation at 488 nm, using a Perkin Elmer Luminescence Spectrometer LS50B. 5 different concentrations of eGFP were measured in the presence of 8 different concentrations of **AdoCbl** (0, 0.25, 0.5, 1, 2, 3, 4, 6 µM) in order to generate a calibration curve (see main part, Fig. 4).

Table S2. Comparison of EC_{50} and A_M values for the investigated corrinoids. Values of EC_{50} (concentration of corrinoid ligand when half of the inhibition is observed) and of A_M (maximal extent of inactivation) obtained from fluorescence and densitometry analysis for **AdoCbl**, **AdoRhbl** and **CNCbl**.

**A comparison between different B_{12} variants on their effect on the *E. coli btuB* riboswitch**				
**Cobalamin type**	**Method of Analysis**	**EC_{50} (µM)**	**Standard Error for EC_{50} value**	**A_M (%)**
AdoCbl	Fluorescence	0.8	+/- 0.1	82
AdoCbl	Densitometry	3.1	+/- 0.3	82
AdoRhbl	Fluorescence	2.8	+/- 0.7	73
AdoRhbl	Densitometry	1.9	+/- 0.2	78
CNCbl	Fluorescence	31.4	+/- 5.2	63
CNCbl	Densitometry	77.3	+/- 39.2	54

Supplementary chemical formulae

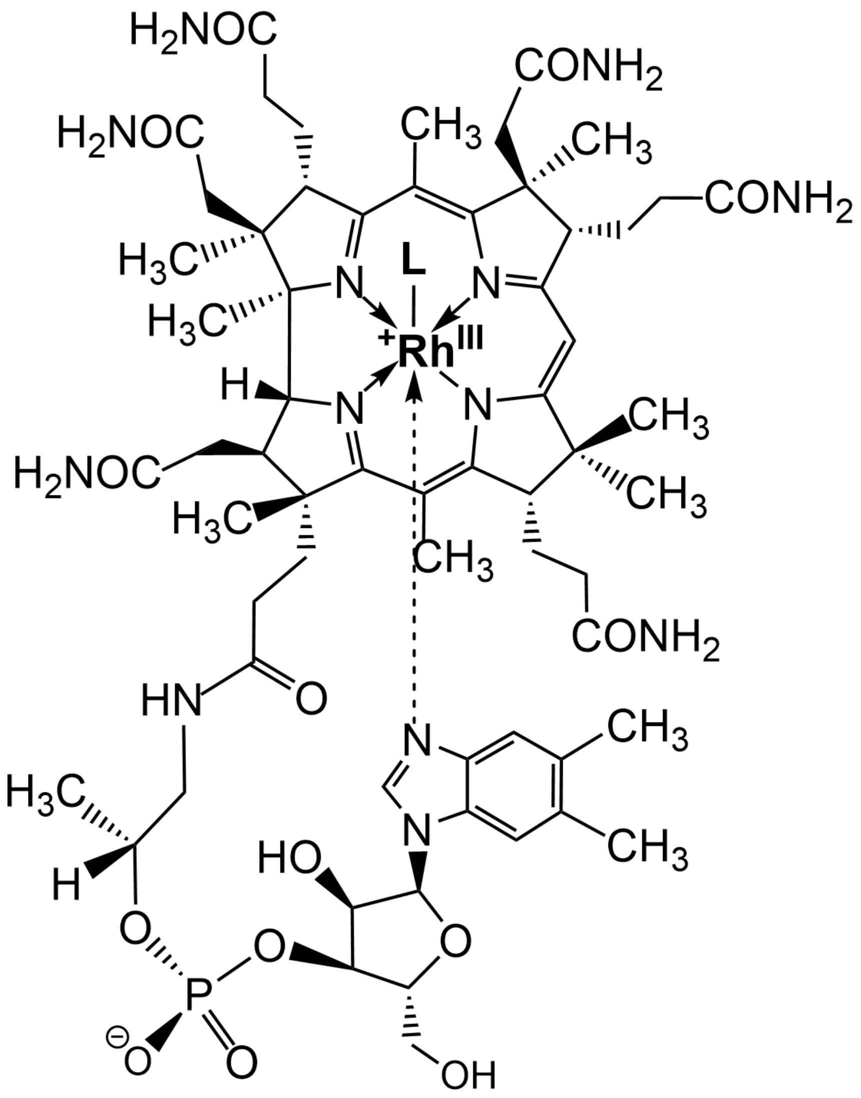

L = Cl: chlororhodibalamin (**ClRhbl**)
L = acetyl: acetylrhodibalamin (**AcRhbl**)

Figure S5a. Supplementary chemical formulae of chlororhodibalamin (**ClRhbl**) [1] and acetylrhodibalamin (**AcRhbl**) [7]

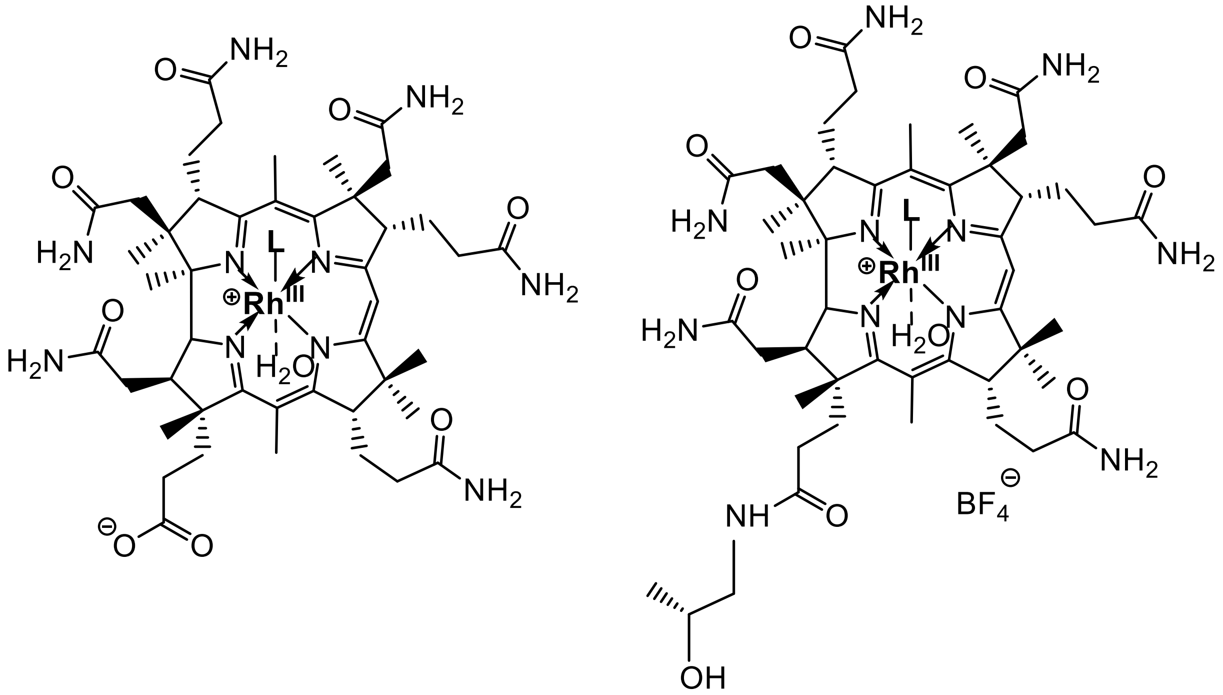

L = CH_3: methylrhodibyrate (**MeRhby**)
L = Ado : adenosylrhodibyrate (**AdoRhby**)

L = Ado :
adenosylrhodibinamide (**AdoRhbi**)

Figure S5b. Chemical formulae (left) of the rhodibyrates methylrhodibyrate (**MeRhby**) [8] and adenosylrhodibyrate (**AdoRhby**) [2, 9] and (right) of adenosylrhodibinamide tetrafluoroborate (**AdoRhbi**).[8]

References

[1] F. J. Widner, C. Kieninger, K. Wurst, E. Deery, M. J. Warren, B. Kräutler, *Synthesis* **2021**, *53*, 332–337.

[2] F. J. Widner, A. D. Lawrence, E. Deery, D. Heldt, S. Frank, K. Gruber, K. Wurst, M. J. Warren, B. Kräutler, *Angew. Chem. Int. Ed.* **2016**, *55*, 11281-11286.

[3] M. Ruetz, A. Shanmuganathan, C. Gherasim, A. Karasik, R. Salchner, C. Kieninger, K. Wurst, R. Banerjee, M. Koutmos, B. Kräutler, *Angew. Chem. Int. Ed.* **2017**, *56*, 7387-7392.

[4] A. Nahvi, J. E. Barrick, R. R. Breaker, *Nucl. Acids Res.* **2004**, *32*, 143-150.

[5] C. V. Franklund, R. J. Kadner, *J. Bacteriol.* **1997**, *179*, 4039-4042.

[6] C. C. Fowler, E. D. Brown, Y. F. Li, *Chem. & Biol.* **2010**, *17*, 756-765.

[7] M. Wiedemair, C. Kieninger, K. Wurst, M. Podewitz, E. Deery, M. D. Paxhia, M. J. Warren, B. Kräutler, *Helv Chim Acta* **2023**, *106*, e202200158.

[8] F. J. Widner, C. Kieninger, B. Kraeutler, *J. Porph. Phthal.* **2025**, *29*, 408-417.

[9] M. Ruetz, R. Mascarenhas, F. Widner, C. Kieninger, M. Koutmos, B. Kräutler, R. Banerjee, *Biochemistry* **2024**, *63* 1955–1962.
